# Dynamic gene network reconstruction from gene expression data in mice after influenza A (H1N1) infection

**DOI:** 10.1186/2043-9113-1-27

**Published:** 2011-10-21

**Authors:** Konstantina Dimitrakopoulou, Charalampos Tsimpouris, George Papadopoulos, Claudia Pommerenke, Esther Wilk, Kyriakos N Sgarbas, Klaus Schughart, Anastasios Bezerianos

**Affiliations:** 1 School of Medicine, University of Patras, Patras 26500, Greece; 2 Department of Electrical and Computer Engineering, University of Patras, Patras 26500, Greece; 3 Department of Infection Genetics, Helmholtz Centre for Infection Research, Inhoffenstr. 7, D-38124 Braunschweig, Germany; 4 University of Veterinary Medicine Hannover, Buenteweg 2, D-30559 Hannover, Germany

**Keywords:** Gene Regulatory Network, Time Varying Dynamic Bayesian Network, Immune System, Influenza A

## Abstract

**Background:**

The immune response to viral infection is a temporal process, represented by a dynamic and complex network of gene and protein interactions. Here, we present a reverse engineering strategy aimed at capturing the temporal evolution of the underlying Gene Regulatory Networks (GRN). The proposed approach will be an enabling step towards comprehending the dynamic behavior of gene regulation circuitry and mapping the network structure transitions in response to pathogen stimuli.

**Results:**

We applied the Time Varying Dynamic Bayesian Network (TV-DBN) method for reconstructing the gene regulatory interactions based on time series gene expression data for the mouse C57BL/6J inbred strain after infection with influenza A H1N1 (PR8) virus. Initially, 3500 differentially expressed genes were clustered with the use of k-means algorithm. Next, the successive in time GRNs were built over the expression profiles of cluster centroids. Finally, the identified GRNs were examined with several topological metrics and available protein-protein and protein-DNA interaction data, transcription factor and KEGG pathway data.

**Conclusions:**

Our results elucidate the potential of TV-DBN approach in providing valuable insights into the temporal rewiring of the lung transcriptome in response to H1N1 virus.

## Background

It is now well established that the study of biological complexity has shifted from gene level to interaction networks and this shift from components to associated interactions has gained increasing interest in network biology. *Gene Regulatory Networks *(GRNs) depict the functioning circuitry in organisms at the gene level and represent an abstract mapping of the more complicated biochemical network which includes other components such as proteins, metabolites, etc. Understanding GRNs can provide new ideas for treating complex diseases and offer novel candidate drug targets. A commonly accepted top-down approach is to reverse engineer GRNs from experimental data generated by microarray technology [1-5].

Early computational approaches for inferring GRNs from gene expression data employed classical methods. Boolean network modeling considers the gene expression to be in a binary state (either switched on or off), and display via a Boolean function the impact of other genes on a specific target gene [6]. Nevertheless, the intermediate levels of gene expression are neglected, thus resulting in information loss. Moving forward, Bayesian networks (BN) utilize probability calculus and graph theory and model GRNs as directed acyclic graphs where the nodes represent genes and the edges between nodes represent regulatory interactions, based on the conditional dependencies extracted from the data. Despite their ability to deal with noisy input, they ignore the temporal dynamic aspects that characterize GRN modeling [7]. To cope with that, the Dynamic Bayesian Networks (DBN) evolved feedback loops to incorporate the temporal aspects of regulatory networks; however the computational cost for estimating the conditional dependencies remains high when the number of genes is large [8,9]. Also, linear additive regulation models managed to identify certain linear relations in regulatory systems but failed to attribute the nonlinear dynamics features [10].

Recently, several techniques have been developed for the mathematical modeling of the dynamics of gene-gene interactions from time series expression data, such as differential equation based models [11-14], state space models [15,16], vector autoregressive (VAR) models [17,18] and information theoretic models [19]. However, the resulting network structures are static, with time-invariant topology among the defined set of nodes. Therefore, these network structures can be characterized 'dynamic' only in the sense that they model dynamical systems. It still remains a challenging task to model in a quantitative manner the dynamic character of biological networks, which in turn appear, based on the latest studies, not to be static networks with invariant topology but are rather context-dependent and systematically rewired over time. These time or context dependent functional circuitries are referred as time varying biological networks [20-26].

Our study focuses on depicting the temporal dynamics of the lung transcriptome after perturbation of the biological system by an infection with influenza A virus. Intensive research has already been performed in analyzing the viral virulence factors and genetic host factors contributing to disease development and outcome [27-31]. The innate immune response system is the first line of defense against pathogens and more fast acting in comparison to adaptive immune response. However, little knowledge exists about the influence of specific genes or gene interactions that contribute to the susceptibility or resistance to influenza infections. Our effort was to provide the directed time evolving network structures underlying the innate immune regulatory mechanism, with temporal resolution up to every single time point based on the time series measurements of the nodal state. Our goal was to provide evidence that the immune response mechanism undergoes significant 'tuning' during the first 5 days after pathogen invasion and present these shifts through serial snapshots, each one depicting the evolutionary steps of gene interplay. In our approach we applied the Time Varying Dynamic Bayesian Networks (TV-DBNs) on a time series microarray dataset obtained from the lungs of C57BL/6J mice infected with a mouse-adapted influenza A (H1N1) virus. It has already been shown, that time varying network approaches like TV-DBNs [26] have provided valuable insights in depicting the transitional changes in yeast cell cycle or studies like Song et al. [32] that successfully exhibited the stages of developmental cycle of *D. melanogaster*. The TV-DBNs offer the ability to overcome limitations of other approaches like the structure learning algorithms for Dynamic Bayesian networks [7], that depict dynamic systems with fixed node dependencies or other approaches like [33], where a static network is constructed as a start point and then time dependencies are detected.

One important aspect of our research was to bring together clustering and inferring networks from time series data. From the computational point of view, the number of estimated relationships in the network is significantly reduced by defining relationships on cluster level [34-36], thus network inference becomes more feasible. Also, recent studies have characterized biological networks as modular, with modules defined as groups of genes, proteins or other molecules participating in common subcellular processes [37,38]. Based on that concept, clusters of co-regulated genes can also be considered as abstractions of modules, since the underlying idea is that co-regulated genes are usually functionally associated. In our approach, we aim at defining relationships between clusters, rather than gene-to-gene relationships, which in turn can be regarded as special cases of clusters (i.e. with each gene defining its own cluster).

Summarizing, the present reverse engineering approach consists of four steps: (1) data selection, (2) clustering for obtaining centroids, (3) parameter tuning and generation of Time Varying Dynamic Bayesian Networks based on the time series experimental expression profiles of cluster centroids and (4) evaluation of the resulting networks with respect to topological measures as well as with available biological knowledge.

## Methods

### Data

C57BL/6J mice were infected with a mouse-adapted influenza A virus (PR8), RNA was prepared from whole lungs and processed for hybridization on Agilent 4 × 44 k arrays. Three replicates, from three individually infected mice, were taken for each time point after infection (1, 2, 3, 4, 5 days) and from three mock-infected mice (day 0) (Pommerenke C et al.: Global transcriptome analysis in influenza-infected mouse lungs reveals the kinetics of innate immune responses, infiltrating T cells, and formation of tertiary lymphoid tissues, submitted). All experiments in mice were approved by an external committee and according to the national guidelines of the animal welfare law in Germany ('Tierschutzgesetz in der Fassung der Bekanntmachung vom 18. Mai 2006 (BGBl. I S. 1206, 1313), das zuletzt durch Artikel 20 des Gesetzes vom 9. Dezember 2010 (BGBl. I S. 1934) geändert worden ist.'). The protocol used in these experiments has been reviewed by an ethics committee and approved by the 'Niedersächsiches Landesamt für Verbraucherschutz und Lebensmittelsicherheit, Oldenburg, Germany', according to the German animal welfare law (Permit Number: 33.9.42502-04-051/09). Preprocessing steps of the raw data comprised background correction [39], quantile normalization, probe summarization, and log2 transformation using the R environment and additional packages from Bioconductor [40].

Subsequently, we used the GEDI toolbox [41] in order to identify the differentially expressed gene probes and after applying t-test with p-value < 0.05 (FDR adjusted), 3500 genes were maintained. We examined our gene list with the use of Database for Annotation, Visualization, and Integrated Discovery (DAVID) functional annotation tool [42] for over-represented biological process Gene Ontology terms (results shown in Table 1).

**Table 1:** GO enrichment analysis

GO Biological Process Term	Percentage (%)	P-Value
GO:0002376:immune system process	7.5	7.45E-31

GO:0050896:response to stimulus	15.2	1.83E-11

GO:0009987:cellular process	48	1.22E-06

GO:0051704:multi-organism process	2.7	1.54E-06

GO:0016265:death	3.2	0.001708142

GO:0040011:locomotion	2.3	0.005231518

GO:0008152:metabolic process	35.4	0.036706589

GO:0016043:cellular component organization	10	0.037186976

GO:0032502:developmental process	14.2	0.061325344

Biological Process GO enrichment analysis of the 3500 genes included in our dataset. The analysis was implemented with DAVID Bioinformatics Resources functional annotation tool. 1429 out of the 3500 genes are not yet characterized with regard to biological process GO terms.

### Clustering

Clustering and gene network inference methods are usually developed independently. However, it is widely accepted that deep relationships exist between the two and their implementation in a unified manner overcomes the limitations posed by each method. A challenging task in gene network reconstruction is that the number of genes is so large; hence network modeling based on a limited amount of data becomes too complex. The general opinion is that the amount of data required for GRN modeling increases approximately logarithmically with the number of genes [43]. However, it is difficult to specify the experimental data requirements more precisely since many more factors influence the network inference performance. Also, the quality of an inferred model depends on the quality of the given data; the number of time points (in case of time series data), the observation duration and the interval between subsequent measurements might lead to less informative data and thus hamper a reliable GRN reconstruction. In order to overcome the limitations posed by the large number of genes, some types of dimensionality reduction of the network are necessary. Based on the fact that genes with similar expression profiles are considered to be co-regulated, reconstructing networks at cluster level is a realistic and statistically advantageous approach, since the dimensions of the cluster-based networks become significantly lower. From a system theoretic perspective, coarse graining of expression profiles means removing redundant information. Therefore, one reasonable approach is to group genes into clusters by means of a clustering technique and then use the cluster centroids or cluster representatives as input for subsequent modeling [34]. Nevertheless, it should be noted that clustering results are often characterized as ambiguous, since they depend on the clustering method, the selection of distance metric and initialization parameters. In our study, we chose to cluster the temporal profiles with the use of k-means algorithm due to its simplicity and fast speed in processing large datasets. The clustering process was repeated more than 100 times using random initialization, with Euclidean metric as distance measure. We implemented the Euclidean distance as a similarity measure, in order to detect similar expression trends (positive linear correlation) i.e. simultaneous up or down regulated expression levels. From the biological perspective, it is considered more important to identify the relative up/down regulation of expression profiles than the amplitude absolute expression changes [44]. Furthermore, the optimal number of clusters was appointed both by means of the Dunn index [45] as well as by GO enrichment analysis. Therefore, the obtained cluster centroids can be rightfully employed as input in the TV-DBN algorithm.

**Figure 1:**
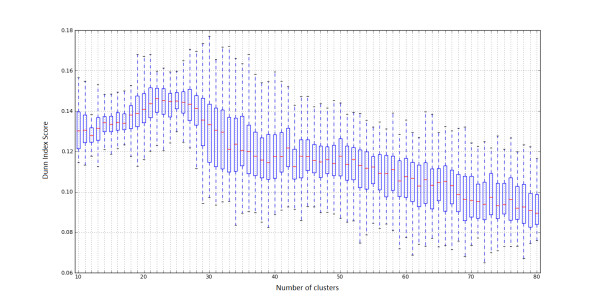
**Dunn Index results**. Boxplot with Dunn Index results for k-means clustering. The x-axis represents the cluster number, while the y-axis represents the Dunn's cluster validity index scores. The experiment was repeated 100 times and the maximal Dunn Index score values were observed in the range of 19-36 cluster size.

In particular, we applied k-means clustering algorithm at the data with the cluster number ranging between 10 and 80. We selected this range, so that the resulting cluster number is both indicative enough of the size of our dataset as well not so large, avoiding so over-fitting that leads to poor predictive power. We employed Dunn index, a performance measure used for comparing different clustering results, in order to check the range of cluster number that gives dense and well separated clusters. This index is defined as the ratio between the minimal inter-cluster distance to maximal intra-cluster distance. As intra-cluster distance the sum of all distances to their respective centroid was calculated, while the inter-cluster distance was defined as the distance between centroids. According to the internal criterion of the index, clusters with high intra-cluster similarity and low inter-cluster similarity are more desirable. The maximal Dunn index score values were observed between 19-36 clusters as can be seen in Figure 1. However, the final number of clusters was estimated after examining the clusters, assessed from the best clustering result in terms of maximal Dunn index scores, with regard to Gene Ontology biological process terms, so that the obtained clusters are biologically sensible and functionally coherent. In detail, we analyzed our clusters, with the use of DAVID functional annotation tool at level 3, for enriched GO terms, the percentage of genes related to that term and the corresponding EASE score, which is a modified Fisher Exact p-value and concluded that 35 clusters was the optimal number (the gene members of every cluster are displayed in additional file 1). We chose to check clusters at level-3 in order to avoid the impact of the broadest terms or the most specific ones on the enrichment analysis. It is worth mentioning that the majority of our genes (1429 genes) are not yet fully characterized by GO terms, thus our clusters leave space for further exploration. Therefore, we characterized our clusters based on the rest genes, fully described in terms of GO terms (additional file 2). We found that 13 clusters are characterized by terms associated to immune response, whereas the rest are mainly involved in metabolic process and system development.

### Time Varying Dynamic Bayesian Network Modeling

A Time Varying Dynamic Bayesian Network (TV-DBN) is a model of stochastic temporal processes based on Bayesian networks [26]. It represents relations between the state of a variable at one time point and the states of a set of variables at previous time points.

Given a set of time series in the form of

$$X^{t}:={\left(X_{1}^{t},...,X_{p}^{t}\right)}^{T}\in R^{p}$$

where *t *is a time in the timeseries, X^t ^is a vector of the values of p variables at time *t*, a TV-DBN models relations as:

$$X^{t}=A^{t}\cdot X^{t-1}$$

where *A^t ^*∈ *R*^*p *× *p *^is a matrix of coefficients that relate the values at *t-1 *to those of time *t*. The non-zero elements of A^t ^form the edge set of the network for time *t*.

In our experiments, each cluster was a variable of the model and its centroid gave the time series values. Thus, the resulting networks relate the expression levels of all clusters at previous time point to the expression levels of each cluster at each time point. In order to calculate the network structures, it is assumed that they are sparse and vary smoothly across time; therefore successive networks are likely to share common edges. The problem of estimating the networks is decomposed into smaller, atomic optimizations, one for each node *i (i = 1...p) *at each time point t* (t* = 1...T):

$${\hat{A}}_{i\cdot }^{t*}=\underset{A_{i\cdot }^{t*}\in R^{1\times n}}{argmin}\frac{1}{T}\sum_{t=1}^{T}w^{t*}(t){\left(x_{i}^{t}-A_{i\cdot }^{t*}x^{t-1}\right)}^{2}+\lambda {\left\|A_{i\cdot }^{t*}\right\|}_{1}$$

where *λ *is a parameter for the **ℓ_1_**-regularization term, which controls the number of non-zero entries in the estimated ${\hat{A}}_{i\cdot }^{t*}$, and hence the sparsity of the networks; $w^{t*}\left(t\right)$ is the weighting of an observation from time t when estimating the network at time t*, and is defined as:

$$w^{t*}\left(t\right)=\frac{K_{h}\left(t-t*\right)}{\sum_{t=1}^{T}K_{h}\left(t-t*\right)}$$

where:

$$K_{h}\left(t\right)=\exp\left(-\frac{t^{2}}{h}\right)$$

is a Gaussian RBF kernel function and *h *is the kernel bandwidth. The above optimization is transformed further by scaling the covariates and response variables by

$$\sqrt{w^{t*}\left(t\right)}$$

i.e. ${\tilde{x}}_{i}^{t}\leftarrow \sqrt{w^{t*}\left(t\right)}x_{i}^{t}$ and ${\tilde{x}}^{t-1}\leftarrow \sqrt{w^{t*}\left(t\right)}x^{t-1}$

The optimization is then solved using the shooting algorithm [46], which iteratively updates one entry of A_i _while holding all other entries fixed. The kernel bandwidth *h *affects the contribution of temporally distant observations. A high value results in all observations contributing equally to each time point, while a small value narrows the effect to only the immediately previous time point. For our experiments, we selected *h *so that the weighting of observations 2 days away from each time point is higher than exp(-1).

$$K_{h}\left(2\right)=\exp\left(-\frac{2^{2}}{h}\right)>\exp\left(-1\right)$$

The **ℓ_1_**-regularization term *λ *affects the sparsity of the resulting networks and controls the tradeoff between the data fitting and the model complexity. In order to set the appropriate value to *λ*, we employed the Bayesian Information Criterion (BIC) [32] and the largest BIC score value was detected when *λ *was set to 0.1. An implementation of the estimation algorithm was created in Python programming language, using the NumPy and Scipy libraries.

## Results and Discussion

The current study proposes a systems biology approach to analyze the dynamic behavior of the lung transcriptome to H1N1 infection from stimulus-response data from perturbation experiments. This system can be regarded as a specific stimulus-induced perturbed biological system. In particular, we present an implementation of Time Varying Dynamic Bayesian Networks on time series gene expression data of murine C57BL/6J inbred strain after infection with H1N1 (PR8) virus. Our reverse engineering approach combines clustering techniques and network inference methods, in order to map the dynamic gene regulatory kinships occurring at various time points after infection, thus displaying the response of the lung transcriptome after an environmental stimulus. However, the low time resolution of data imposed significant constraints in analysis and modeling. Therefore, we permuted our analysis by defining the regulatory effects on cluster level in order to achieve some kind of dimensionality reduction. The resulting five TV-DBNs, each one representing the GRN at a specific time point (day p.i.), were evaluated with topological metrics as well as with available interactome data. Also, we checked whether known gene-to-gene relationships could be retrieved from our cluster based approach.

### Topological analysis of Regulatory Networks

**Figure 2:**
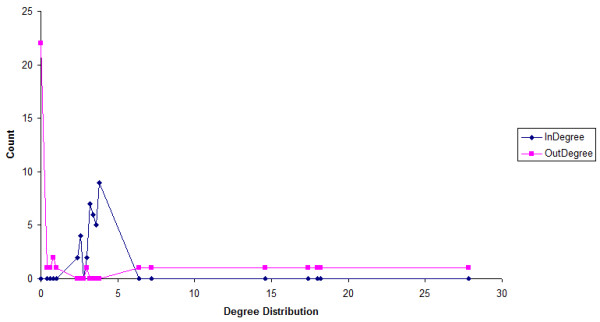
**Degree Distribution**. Indegree and outdegree distribution averaged over 5 time points. The x-axis represents the indegree/outdegree score, while the y-axis depicts the total number of clusters.

**Figure 3:**
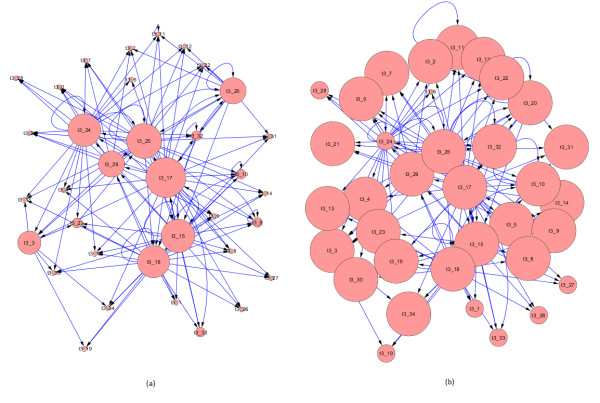
**Network Graph Structures**. Network graph structures of the resulting TV-DBNs. Two indicative networks with different sized nodes from time point 3 are displayed, in terms of (a) outdegree score and (b) indegree score. Each node represents the time (t) of the respective network and the corresponding cluster number.

The first goal in our analysis was to explore the topological characteristics of the five TV-DBNs. Thus, we conducted local topology analysis in order to identify hub or bottleneck clusters/nodes that could serve as the key regulators at every time point. For this purpose we used Hubba server [47] and calculated several network topology metrics such as degree (D), bottleneck (BN), edge percolated component (EPC), Maximum Neighborhood Component (MNC) and Density of Maximum Neighborhood Component (DMNC). Also, we used the Cytoscape plugins [48] for network analysis and measured the indegree, outdegree and betweenness centrality metrics. Indegree is the count of the number of interactions directed to the node, and outdegree is the number of interactions that the node directs to other nodes. Betweenness centrality measures on how many shortest paths a node, between other nodes, occurs. It has been shown that metrics like the aforementioned improve the identification of essential nodes in networks. For example, betweenness centrality correlates closely with essentiality, exposing critical nodes that usually belong to the group of scaffold proteins or proteins involved in crosstalk between signaling pathways (called *bottlenecks*) [49]. This metric has also been proposed in the new paradigm of network pharmacology as a good feature for investigating potential drug targets [50]. The results are displayed in Table 2 where we detected the 'top scorer' clusters for every metric and for each TV-DBN separately. With regard to betweenness centrality, the majority of the clusters are related to immune response, with the exception of clusters 20, 25, 33 which are related with cell-cell adhesion, regulation of cellular process and cellular macromolecule metabolic process. The scene is repeated with regard to BN metric, where all top scorer clusters are immune response related, with the cluster 20 as exception. Bottlenecks are network nodes with key connector role in the network and have many 'shortest paths' going through them. The MNC metric displays similar results with betweenness centrality, with cluster 0 detected by MNC but not by betweenness centrality. Also, the EDC metric has similar results with MNC and betweenness centrality with few variations, especially in the ranking of the top scorer clusters. Interesting results can also been extracted from the out- and in-degree scores. All top scorer outdegree clusters can be considered as the key '*regulators' *whereas the top indegree clusters as the significantly '*regulatee' *clusters. As seen, the majority of outdegree clusters are immune response related in terms of KEGG pathways [51] (Table 3), but one can observe that at day 1 post infection (p.i.) cluster 3 (GO: cellular macromolecule metabolic process) appears as significant regulator and then vanishes from the highest rank positions. Also, clusters 17 and 18 lose their central role especially at day 4 p.i. where clusters like 25 (GO: system development) are recruited. With respect to indegree metric, the majority of clusters displayed similar scores with the top 5 presented clusters, whereas the outdegree top 5 clusters had significant score value differences with the rest clusters. We also plot the histogram of indegree and outdegree (averaged across time) for the time-varying networks in Figure 2. The outdegrees seem to follow a scale free distribution, which means that few clusters (regulators) regulate a lot of clusters, whereas the indegree distribution is very different from that of the outdegree and indicates that most clusters are controlled by a few clusters. The average indegree score per cluster centroid node is 3.23, which is indicative of the underlying model complexity. This value could be regarded as high if gene-gene relationships were considered, but the presented approach is based on cluster centroid expression profiles, which in turn represent the expression trend of sets of genes and therefore the indegree term should be interpreted from a different perspective. In Figure 3, we display an indicative example of the outdegree and indegree distribution of clusters with different sized nodes at day 3 p.i. The directed interactions display the snapshot of the regulatory relationships among the gene clusters at the specific time point. It is evident that few clusters have high outdegree scores, while the majority of clusters have similar scores with respect to indegree metric (the highest scores are presented in Table 2). These findings are well consistent, on gene level, with the biological observations that most genes are controlled only by a few regulators.

**Table 2:** Top Scorer Clusters

					Time Point (day p.i.)																				
*Topological Metric*	*1(day p.i.)*					*2(day p.i.)*					*3(day p.i.)*					*4(day p.i.)*					*5(day p.i.)*				
***Rank***	***1***	***2***	***3***	***4***	***5***	***1***	***2***	***3***	***4***	***5***	***1***	***2***	***3***	***4***	***5***	***1***	***2***	***3***	***4***	***5***	***1***	***2***	***3***	***4***	***5***

**Hubba MNC**	17	18	24	15	0	17	18	15	24	25	17	25	15	18	24	17	25	15	24	18	25	24	15	17	18
**Hubba EPC**	17	18	24	15	20	17	24	15	18	25	17	25	15	24	18	17	25	15	24	18	25	15	24	18	17

**Hubba DMNC**	0	10	4	6	7	11	14	20	32	0	2	11	12	22	31	31	0	4	10	7	0	11	22	28	31
**Hubba Degree**	17	18	24	15	0	17	18	15	24	25	17	15	25	18	24	17	25	15	18	24	25	24	15	18	17

**Hubba BN**	17	18	15	-	-	17	15	-	-	-	18	17	15	24	-	17	18	15	24	-	18	24	15	17	20
**Indegree**	10	11	7	9	22	11	14	9	32	17	10	11	32	8	9	10	11	24	23	32	10	11	14	23	9

**Outdegree**	17	18	24	15	3	17	18	15	24	25	17	15	25	18	24	17	25	15	24	18	25	15	24	18	17
**Betweenness Centrality**	17	18	15	24	33	17	18	15	20	25	17	18	29	15	25	17	25	18	23	15	18	17	25	15	23

Clusters were evaluated in every time point with several topological metrics as defined in Hubba analyzer. Also, the indegree, outdegree and betweenness centrality scores were calculated with the use of Cytoscape plugins. We display the top 5 clusters (with descending rank order) at every time point with the highest scores in every metric, with the exception of BN metric where only few clusters had score > 0.

**Table 3:** KEGG Pathway analysis

Outdegree/Betweenness Centrality			
**Cluster**	**KEGG pathway**	**Percentage**	**P-value**

3	no pathway		

15	B cell receptor signaling pathway	11.5	8.00E-03

17	RIG-I-like receptor signaling pathway	21.1	6.30E-06
	Cytosolic DNA-sensing pathway	15.8	5.30E-04
	Toll-like receptor signaling pathway	10.5	6.70E-02

18	Natural killer cell mediated cytotoxicity	16.7	2.60E-03
	Graft-versus-host disease	11.1	4.00E-02
	Allograft rejection	11.1	4.00E-02

20	drug metabolism	10.8	1.30E-03

23	Jak-STAT signaling pathway	6.0	9.60E-03
	Lysosome	4.8	2.80E-02
	Cell adhesion molecules (CAMs)	4.8	5.30E-02

24	Cytokine-cytokine receptor interaction	22.7	4.50E-05
	Chemokine signaling pathway	18.2	5.90E-04
	NOD-like receptor signaling pathway	13.6	1.70E-03
	Cytosolic DNA-sensing pathway	9.1	5.60E-02
	Hematopoietic cell lineage	9.1	8.50E-02
	Toll-like receptor signaling pathway	9.1	9.90E-02

29	Proteasome	6.3	1.00E-03
	Apoptosis	4.8	5.40E-02
	Toll-like receptor signaling pathway	4.8	6.80E-02

33	Aldosterone-regulated sodium reabsorption	3.4	7.40E-03

**Indegree**			

**Cluster**	**KEGG pathway**	**Percentage**	**P-value**

7	DNA replication	9.7	4.60E-09
	Mismatch repair	5.6	9.40E-05

8	Apoptosis	3.2	1.40E-02
	p53 signaling pathway	2.4	6.00E-02

9	Chemokine signaling pathway	8.9	8.80E-03
	Jak-STAT signaling pathway	6.7	5.20E-02

10	Antigen processing and presentation	8.7	2.40E-05
	Allograft rejection	7.2	7.20E-04
	Endocytosis	8.7	1.00E-03
	Viral myocarditis	5.8	5.90E-03

11	Complement and coagulation cascades	8.2	3.10E-05
	Cytokine-cytokine receptor interaction	9.6	1.70E-03

14	Natural killer cell mediated cytotoxicity	13.5	5.00E-08
	T cell receptor signaling pathway	8.5	8.70E-04
	Primary immunodeficiency	5.4	5.70E-03
	Cell adhesion molecules (CAMs)	8.1	2.80E-03
	Leukocyte transendothelial migration	6.8	6.80E-03
	Cytokine-cytokine receptor interaction	8.1	1.90E-02
	Cell adhesion molecules (CAMs)	3.8	1.70E-02
	Cytokine-cytokine receptor interaction	8.1	1.90E-02
	Cell adhesion molecules (CAMs)	3.8	1.70E-02

22	DNA replication	3.4	2.30E-03
	Cytokine-cytokine receptor interaction	5.2	3.80E-02

23	Jak-STAT signaling pathway	6.0	9.60E-03
	Cell adhesion molecules (CAMs)	4.8	5.80E-02

24	Cytokine-cytokine receptor interaction	22.7	5.40E-05
	Chemokine signaling pathway	18.2	5.90E-04
	NOD-like receptor signaling pathway	13.6	1.70E-03

32	Cytokine-cytokine receptor interaction	19.6	2.00E-09
	NOD-like receptor signaling pathway	8.9	5.30E-05
	Toll-like receptor signaling pathway	8.9	1.30E-04

All top scorer clusters, with regard to indegree, outdegree and betweenness centrality metrics, were checked for enriched KEGG pathways.

**Figure 4:**
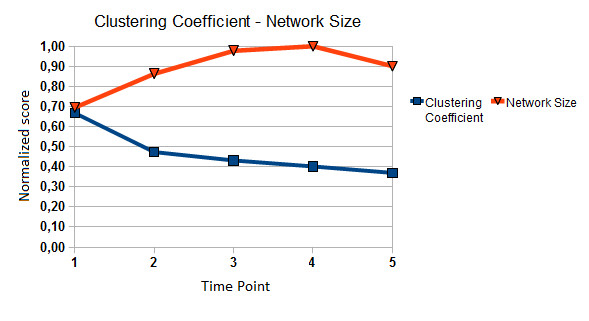
**Network Size/Local Clustering Coefficient**. Plot of two network statistics (network size, clustering coefficient) as functions of time line. It is obvious that network size evolves in a very different way from the local clustering coefficient.

In Figure 4, two different statistics, network size and average local clustering coefficient, of the reversed engineered cluster-based regulatory networks are plotted as a function of the five time phases. Network size, defined as the number of edges, depicts the overall connectedness of the network, while the average local clustering coefficient, as defined by [52], measures the average connectedness of the neighborhood local to each node. Both statistics have been normalized to the range between 0[1] for comparison reasons. It is apparent that the network size and the average local clustering coefficient display completely different trajectories during the defense response against the virus. On one hand, the network size is continually increasing, displaying peak value at day 4 p.i. and then slightly drops. On the other hand, the average local clustering coefficients of the TV-DBNs drop sharply after day 1 p.i. and stay low until the fifth day after infection. One possible explanation is that the clusters of co-expressed genes have a more fixed and specific role at the beginning of the battle against the pathogen and therefore interact with fewer clusters; however, the genes show an expanded functionality repertoire in the next critical days in order to serve the needs for response against the virus. A further hypothesis is that in interactome exist few key modules/clusters (hubs) that initiate most of the other modules to be activated in the beginning of response, and this feature is lost at the late time phases, where the 'hub-ness' identity is diffused in more modules apart from the key ones. After all, the viral load develops gradually during the first days of infection, displaying a peak on day 2 p.i., which might be the critical threshold for the onset of immune response.

### Interactome analysis with Protein-Protein and Protein-DNA Interaction data

**Figure 5:**
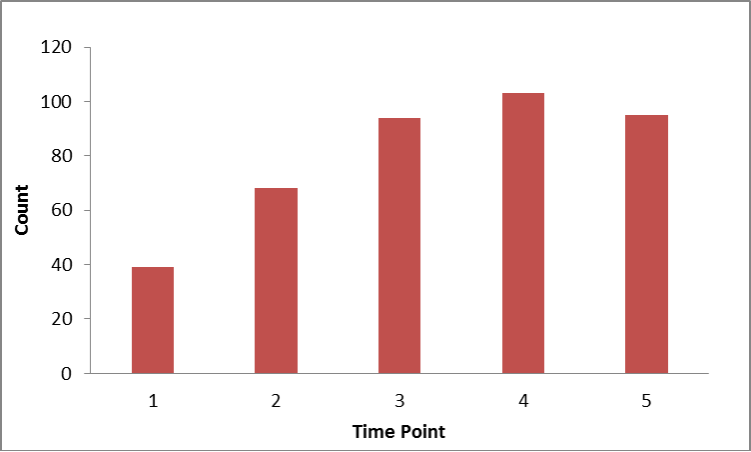
**Size of recovered interactions**. This histogram shows the size of known PPI and protein-DNA interactions recovered per time point. It is apparent that there is an increase in the traced interactions the first 4 days p.i.

An additional aspect in our analysis was to explore the cluster interactome with respect to other types of data such as protein-protein interactions (PPIs) and protein-DNA interactions and display the ability of TV-DBN approach in monitoring the dynamic presence or absence of these interactions over the time course. For this purpose, we downloaded the mouse datasets from InnateDB database [53]. We selected InnateDB because it is a highly curated database that integrates PPI and protein-DNA data from various databases such as DIP, MINT, IntAct, BioGRID and BIND and provides a thorough curation system process for genes/proteins related to innate immune system. In our dataset of a total of 3500 genes, 492 such interaction groups (consisting of more than two genes/proteins) with 381 unique Entrez gene ids were detected (additional file 3). A small fraction (72) of these interaction groups was identified within the members of the clusters, while the rest was shared between clusters. It is apparent in Figure 5 that the traced PPIs and protein-DNA interactions increased abruptly after day 1 p.i. with the peak value at day 4 p.i., probably due to critical viral load development and delayed immune response. This observation is highly correlated with the increase in the network size of the derived TV-DBNs during time evolution, since the interactivity between nodes becomes stronger. It is worth mentioning that the majority of interactions (ranging between 57-69%) detected at each TV-DBN are involved in immune response related pathways like chemokine/cytokines and their receptors, interferon-regulation and interferon-response, TLR signaling pathway, RIG-I-like receptor signaling pathway and others. Despite the limitation posed by the small amount of available PPI and protein-DNA data in our dataset, it is evident that immune response mechanism undergoes significant restructuring the first days after viral invasion and the TV-DBN succeeded in identifying such immune related interactions between different cluster centroid nodes. In Table 4, we list many known PPI and protein-DNA interactions and the precise time point of their occurrence. These observations elucidate the ability of TV-DBNs to provide further hypotheses about the time snapshots that protein-protein and protein-DNA interactions take place.

**Table 4:** Timeline of PPI/Protein-DNA interactions

A	B	C	D	E	PPI/Protein-DNA interaction	
●	●				Relb	Cxcl13

●	●	●	●	●	Nfkb2	Cxcl13

●	●	●	●	●	Nfkbiz	Il6

		●	●	●	Bcl3	Cyld

●	●	●	●	●	Stat1	Gm9706

		●	●	●	Prkcz	Junb

●	●	●	●	●	Cxcl10	Cxcr3

●	●	●	●	●	Stat1	Cxcl10

●	●	●	●	●	Stat2	Cxcl10

●	●	●	●	●	Irf9	Cxcl10

			●	●	Plcg2	Spnb2

●	●	●			Tlr2	Tlr6

●					Ncor1	Cxcl10

	●	●	●	●	Stat4	Ifng

	●	●	●	●	Tbx21	Ifng

	●	●	●	●	Bid	Gzmb

●	●	●	●	●	Irf1	Gbp2

		●	●		Irf1	Il27

	●	●	●	●	Gpnmb	Pla2g4a

			●	●	Sfpi1	Il1b

●	●				Tbp	Ifng

●	●	●	●	●	Ccl7	Ccr2

●	●				Sfpi1	Cxcl9

●					Cxcl9	Cxcr3

	●	●	●	●	Stat1	Cxcl9

●	●	●	●	●	Lcp2	Vav1

●	●	●	●	●	Ptpn6	Vav1

	●	●	●	●	Ccl4	Ccr5

			●	●	Ncor1	Ccl4

		●	●		Irf1	Il15

●	●	●	●	●	Gzmb	Serpinb9

	●	●	●	●	Dok2	Tek

●	●	●			Rad21	Ifng

			●	●	Ccl2	Ccrl2

●	●	●	●	●	Etv6	Lcn2

●	●	●	●		Ripk	Zbp1

●	●	●	●	●	Irf7	Myd88

●	●	●	●	●	Irf7	Ifnb1

●	●	●	●	●	Stat1	Irf7

	●	●	●	●	Gadd45g	Loc100046823

	●	●	●	●	Irf8	Cxcl9

●	●	●	●		Irf8	Gm9706

●	●	●	●	●	Ccl2	Ccr2

●	●				Junb	Il6

	●	●	●	●	Atf3	Il6

●	●				Runx3	Ifng

			●	●	Ncor1	Ccl2

●					Gzmb	Hopx

●	●	●	●		Irf7	Ifna4

●	●	●	●		Ncor1	Cxcl9

●	●	●	●		Il1rl1	Myd88

Each line in the table corresponds to one PPI or protein-DNA interaction. The bullets indicate the exact day (A: day 1 p.i., B: day 2 p.i., C: day 3 p.i., D: day 4 p.i., E: day 5 p.i.) that the corresponding interaction is present in the resulting network.

Furthermore, we accumulated transcription factor (TF) data from the TFCat database [54], a highly curated catalogue containing proven as well as candidate TFs. In our dataset 104 TFs were identified; 26 of them being TF candidates (data shown in additional file 4). We found that 26% of those TFs are located in hub clusters, e.g. 17, 18, 29 and 33 with high rank in the outdegree metric and contain also three TFs related to immune response such as *Irf7 *in cluster 17, *Irf1 *in cluster 29 and *Bmi1 *in cluster 33. A representative example is cluster 17 that includes in addition to *Irf7 *many other interferon-induced genes like *Ifit1, Ifit2, Ifit3, Ifi44 *and interacts bidirectional (in all time points) with cluster 9, which encompasses a great proportion of interferon-induced genes like *Ifi205, Tgtp, Igtp, Irgm, Ifih1, Isg20*. This observation is consistent with the established role of *Irf7 *as an important protective host response during infection. Irf7 induces the a- and b- interferons, which, in turn, regulate the expression of the interferon-induced genes [55]. Another example is cluster 32 which includes *Atf3 *and regulates, in all time shifts except for day 1, cluster 18 which contains *Ifng*. Other studies have shown that *Atf3 *is recruited to transactivate the ***Ifng ***promoter during early Th1 differentiation [56].

### Pathway gene-gene interaction dynamics

Our networks explicitly depict the cluster inter-relationships at every time serial snapshot. The underlying concept of our method is to reconstruct networks that represent the regulatory effect of a co-expressed gene set A (*regulator*) over another set B of co-expressed genes (*regulatees*) at a specific time point. On gene level, we expect to find the regulators of a gene, belonging to cluster B, in the gene pool of cluster A. Thus, moving forward in our analysis we checked whether TV-DBN approach may recover known gene-to-gene interactions from the derived cluster relationships and we reveal the dynamics of these interactions by displaying the exact time points of their occurrence. One example is the RIG-I-like receptor signaling pathway. A foreign RNA is recognized by a family of cytosolic RNA helicases termed RIG-I-like receptors (RLRs). The RLR proteins include *Rig-I*, *Mda5*, and *Lgp2*, which recognize viral nucleic acids and recruit specific intracellular adaptor proteins to initiate signaling pathways that lead to the synthesis of type I interferon and other inflammatory cytokines, which are important for eliminating viruses [57]. We first, examined if its members were included in clusters that interact in the derived networks (at all time points). Subsequently, we investigated if the direction of these edges reflects the '*regulator-regulatee*' roles on the gene level. In particular, 25 genes (out of the 70 included in the pathway) are included in our dataset and TV-DBN managed successfully to recover all known interactions that are represented in the KEGG database. For example, the TV-DBN algorithm captured the interactions between *Ddx58 *(cluster 10) and *Isg15 *(cluster 17), between *Ddx58 *(cluster 10) and *Trim25 *(cluster 32), between *Irf7 *(cluster 17) and *Ifna2 *(cluster 21), *Ifna4 *(cluster 34), *Ifnab *(cluster 19), *Ifna12 *(cluster 21), *Ifnb1 *(cluster 32) and between *Mapk8 *(cluster 27) and *Mapk9 *(cluster 12) with *Tnf *(cluster 10). Nevertheless, one should bear in mind that the time spacing between gene expression measurements, as has been recorded in our present data set, is fairly large in comparison to the real time at which these interactions occur. Therefore, the current cluster-based networks provide only a very coarse representation of the regulatory effects which could be refined by higher time sampling.

Another important example is the Toll-like receptor signaling pathway. Toll-like receptors (TLRs) are responsible for detecting microbial pathogens and initiating innate immune responses. Upon recognition of the pathogens, TLRs stimulate the rapid activation of innate immunity and induce the production of proinflammatory cytokines and upregulation of costimulatory molecules [58]. In particular, 39 out of the 100 genes of this pathway are part of our differentially expressed dataset. The resulting TV-DBNs showed that the majority of the known interactions, occurring between the 39 members, are identified in the first three days after viral invasion and they fade out in the next days. For example, the interactions among *Tlr1 *(cluster 15), *Tlr2 *(cluster 8) and *Tlr6 *(cluster 14), between *Tlr7 *(cluster 11) and *Myd88 *(cluster 29) as well as between *Pik3r3 *(cluster 33) and *Akt3 *(cluster 8) are observed until day 3 p.i., whereas interactions between *Ifnb1 *(cluster 32) and *Ifnar2 *(cluster 12) and among *Stat1 *(cluster 9), *Cxcl10 *(cluster 17) and *Cxcl9 *(cluster 18) are observed until day 5 p.i.

Finally, we zoomed into the dynamics of NOD-like receptor signaling pathway, where 18 out of the 58 members are included in our dataset. Recently, it was shown that *Nlrp3*, member of the NOD-like receptor family, is activated after influenza virus infection. *Nlrp3 *forms a complex, called inflammasome, with apoptosis associated speck-like protein containing a caspase recruitment domain (ASC) and caspase-1 [59]. Activation of caspase-1 through *Nlrp3 *and ASC is necessary for converting pro-Il1b, pro-Il18 and pro-Il33 into mature cytokines. Il1b and Il18 are potent pro-inflammatory cytokines, and Il33 promotes immune responses mediated by Th2 cells. Our TV-DBNs identified interactions between *Mapk3 *(cluster 26), *Ccl5 *(cluster 32) and *Tnf *(cluster 10) as well as between *Mapk8 *(cluster 27), *Mapk9 *(cluster 12) with *Il6 *(cluster 24) in the first two days, while the interaction between *Casp1 *(cluster 14) and *Il1b *(cluster 32) was traced in days 4 and 5 p.i. It is worth mentioning that the amount of the recovered known gene-gene relationships of our cluster-based approach can offer to biologists novel hypotheses, about the involvement of other genes whose functional role is still unknown, yet belong to the same clusters where the gene-gene interactions were detected.

## Conclusions

Using the TV-DBN method on large scale expression data after an external perturbation of a biological system, such as an infection of the lung with a virus, our proposed approach contributed towards obtaining a deeper understanding of the dynamic changes at the molecular level. We succeeded in detecting several gene-gene interactions known to be important in early host response.

In the near future, more refined network structures will be provided and hidden aspects of the innate immune system will be revealed upon availability of experimental data of more dense time series gene expressions. Thus, the dynamically reconstructed GRNs will be available for monitoring H1N1 disease development and outcome.

## Competing interests

The authors declare that they have no competing interests.

## Authors' contributions

KD conceived of the study, implemented the algorithms, did the interpretation of the results and drafted the manuscript. CT and GP implemented the algorithms and drafted the manuscript. CP contributed to the analysis of the raw data and interpretation of the results. EW contributed to the interpretation of the results. KNS designed the flowchart of the computational aspects of the study and co-ordinated the implementation of the algorithms. KS contributed to the writing of the manuscript and interpretation of results. AB conceived of the study, participated in its design and co-ordination. All authors read and approved the final manuscript.

## Supplementary Material

Additional file 1 **Gene members of 35 clusters**. List of gene members for the 35 clusters (with Entrez gene IDs and short description per gene). Click here for file

Additional file 2 **Biological Process GO enrichment analysis of the 35 clusters**. We examined the derived 35 clusters with respect to biological process GO terms with the use of DAVID Bioinformatics Resources functional annotation tool. Click here for file

Additional file 3 **PPI/Protein-DNA Interaction data**. We downloaded InnateDB protein-protein interaction (PPI) and protein-DNA interaction data and isolated all interaction groups with members included in our dataset. Click here for file

Additional file 4 **Transcription factors**. We downloaded all known and candidate Transcription Factors (TFs) from TFCat database. This table displays all TFs included in our dataset and the cluster in which they are located. Click here for file
