# Supplementary material for: Dynamic gene network reconstruction from gene expression data in mice after influenza A (H1N1) infection
Source: J Clin Bioinforma. 2011 Oct 21;1:27. doi: 10.1186/2043-9113-1-27 (PMC3219564; doi:10.1186/2043-9113-1-27)
Supplement: Additional file 2 — Biological Process GO enrichment analysis of the 35 clusters. We examined the derived 35 clusters with respect to biological process GO terms with the use of DAVID Bioinformatics Resources functional annotation tool. [file 2043-9113-1-27-S2.PDF]

We examined the derived 35 clusters with respect to biological process GO terms with the use of DAVID Bioinformatics Resources functional annotation tool.

cluster number	cluster size	GO terms (biological process-DAVID GO level: 3)	percentage (%)	p-value	number of genes not included in DAVID GO terms
0	86	lipid metabolic process	10.5	2.70E-04	62
1	63	cellular nitrogen compound metabolic process	22.2	4.30E-02	41
		nucleobase, nucleoside, nucleotide and nucleic acid metabolic process	20.6	5.50E-02	
		cellular macromolecule process	27	9.70E-02	84
2	155	macromolecule catabolic process	10.4	9.20E-05	
		cellular catabolic process	11.7	1.60E-04	
		protein metabolic process	22.7	4.10E-04	
		cellular macromolecule process	31.8	2.30E-03	
3	32	cellular macromolecule metabolic process	34.4	4.80E-02	21
4	105	response to stress	14.3	6.10E-03	77
5	68	cell redox homeostasis	5.9	8.80E-04	47
		transport	23.5	2.60E-03	
6	161	cellular macromolecule metabolic process	30.4	7.50E-04	82
		nucleobase, nucleoside, nucleotide and nucleic acid metabolic process	20	8.50E-03	
7	72	response to wounding	9.7	1.90E-03	36
		macromolecule biosynthetic process	26.4	2.30E-03	
8	125	positive regulation of cellular process	13.7	6.20E-03	60
		positive regulation of biological process	14.5	8.83E-03	
		protein metabolic process	19.4	4.30E-02	
9	46	defense response	15.6	4.00E-04	21
10	69	regulation of immune system process	13	2.60E-06	39
		response to other organism	11.6	3.80E-06	
		regulation of response to stimulus	11.6	3.30E-05	

11	73	immune effector process	12.3	7.60E-09	51
		regulation of immune response	12.3	2.00E-07	
		regulation of immune response to stimulus	13.7	1.20E-06	
12	160	anatomical structure morphogenesis	10	3.70E-02	112
13	34	embryonic morphogenesis	14.7	6.20E-03	28
		tissue morphogenesis	11.8	1.30E-02	
14	75	regulation of immune system process	16.2	4.80E-08	30
		leukocyte activation	16.2	8.60E-10	
		lymphocyte activation	14.4	3.70E-09	
		regulation of cellular process	43.2	6.63E-02	
15	26	defense response	23	4.00E-04	16
		response to wounding	19.2	1.50E-03	
16	93	cellular biosynthetic process	21.5	8.00E-03	54
		regulation of nitrogen compound metabolic process	18.3	9.80E-03	
17	20	response to virus	26.3	6.50E-08	12
		response to other organism	26.3	6.20E-06	
18	18	response to organic substance	22.2	3.70E-03	11
		defense response	16.7	3.40E-02	
19	146	response to food	2.1	7.00E-04	141
20	37	cell-cell adhesion	8.1	6.00E-02	30
21	149	response to virus	3.4	3.10E-03	141
22	116	regulation of cell communication	10.3	5.70E-03	67
		cellular macromolecule metabolic process	31.9	2.40E-02	
23	84	response to bacterium	7.1	4.40E-04	64
		defense response	8.3	9.90E-03	
24	22	response to wounding	10.9	3.60E-09	8
		defense response	40.9	2.70E-08	

		taxis	10.2	3.20E-04	
25	77	forebrain development	7.8	1.80E-04	41
		regulation of cellular process	41.6	5.30E-04	
		system development	19.5	3.10E-03	
26	98	regulation of cell size	3.1	9.00E-02	95
27	311	cellular macromolecule metabolic process	27	2.30E-03	163
		cellular nitrogen compound metabolic process	18.6	1.40E-02	
		gene expression	15	2.90E-02	
28	49	cell cycle process	16.3	1.20E-04	24
		nucleobase, nucleoside, nucleotide and nucleic acid metabolic process	34.7	4.40E-03	
29	63	defense response	12.7	1.60E-04	42
30	138	regulation of macromolecule metabolic process	22.5	2.70E-04	69
		regulation of metabolic process	23.5	5.80E-04	
31	178	chromosome organisation	4.5	4.00E-02	151
32	57	defense response	19.3	5.30E-07	30
		response to wounding	15.8	6.80E-06	
		chemotaxis	10.5	1.70E-05	
		response to other organism	10.5	6.50E-04	
33	88	cellular macromolecule metabolic process	36.4	6.10E-03	45
34	406	neurological system process	14.8	2.30E-06	222
		signal transduction	18.3	1.10E-03	
		regulation of cellular process	35.1	3.90E-03	
